# Polyphyly of Asian Tree Toads, Genus *Pedostibes* Günther, 1876 (Anura: Bufonidae), and the Description of a New Genus from Southeast Asia

**DOI:** 10.1371/journal.pone.0145903

**Published:** 2016-01-20

**Authors:** Kin Onn Chan, L. Lee Grismer, Anil Zachariah, Rafe M. Brown, Robin Kurian Abraham

**Affiliations:** 1 Biodiversity Institute and Department of Ecology and Evolutionary Biology, University of Kansas, Lawrence, Kansas, United States of America; 2 Department of Biology, La Sierra University, 4500 Riverwalk Parkway, Riverside, California, United States of America; 3 Beagle, Chandakunnu, Wayanad, Kerala, India; Field Museum of Natural History, UNITED STATES

## Abstract

The Asian Tree Toad genus *Pedostibes*, as currently understood, exhibits a conspicuously disjunct distribution, posing several immediate questions relating to the biogeography and taxonomy of this poorly known group. The type species, *P*. *tuberculosus* and *P*. *kempi*, are known only from India, whereas *P*. *hosii*, *P*. *rugosus*, and *P*. *everetti* are restricted to Southeast Asia. Several studies have shown that these allopatric groups are polyphyletic, with the Indian *Pedostibes* embedded within a primarily South Asian clade of toads, containing the genera *Adenomus*, *Xanthophryne*, and *Duttaphrynus*. Southeast Asian *Pedostibes* on the other hand, are nested within a Southeast Asian clade, which is the sister lineage to the Southeast Asian river toad genus *Phrynoidis*. We demonstrate that Indian and Southeast Asian *Pedostibes* are not only allopatric and polyphyletic, but also exhibit significant differences in morphology and reproductive mode, indicating that the Southeast Asian species’ are not congeneric with the true *Pedostibes* of India. As a taxonomic solution, we describe a new genus, *Rentapia* **gen. nov.** to accommodate the Southeast Asian species.

## Introduction

**Fig 1:**
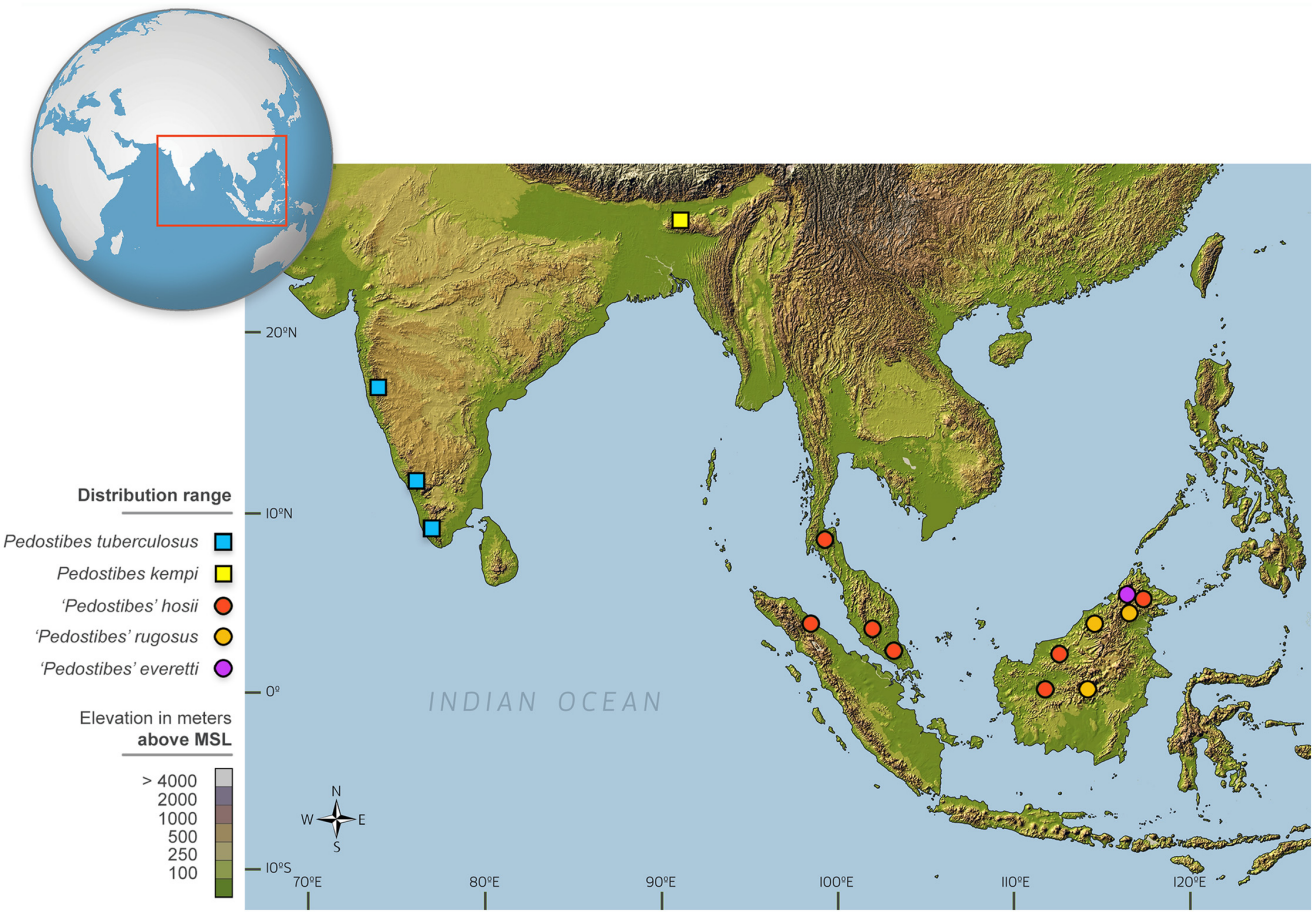
Map showing distribution of species of *Pedostibes* and *Rentapia* gen. nov. Reprinted from http://treehouse-maps.com/ under the CC BY license, with permission from Treehouse Maps, original copyright 2014.

Asian Tree Toads of the genus *Pedostibes* Günther, 1876 comprise five allopatric species, with *P*. *tuberculosus* restricted to the Western Ghats of India [1] and *P*. *kempi*, known only from the Garo Hills in the northeastern part of the Indian subcontinent. Three others, *P*. *hosii*, *P*. *rugosus*, and *P*. *everetti*, are known from Southeast Asia. *Pedostibes hosii* occurs in the Thai-Malay Peninsula from the Isthmus of Kra, southwards to Sumatra and Borneo [2], whereas *P*. *rugosus*, and *P*. *everetti*, are restricted to the island of Borneo [3–5](Fig 1). *Pedostibes hosii* and *P*. *everetti* were originally allocated to the African genus *Nectophryne* [6,7] but were subsequently re-assigned to the genus *Pedostibes* [8]. This re-allocation was justified on the basis of the presence of eight pre-sacral vertebrae and in possessing the coccy articulated by two small, but distinctly separated condyles, which was found common to the Indian *P*. *tuberculosus* and the Southeast Asian species [8].

The type species, *P*. *tuberculosus* is small in size, with females exhibiting a maximum body size of 41.5 mm, (maximum male body size 38.2 mm; this study). The other Indian species, *P*. *kempi*, is known only from the type series, with a reported body size of 34.0 mm [9], and has not been encountered since its original description. The Southeast Asian species, in contrast, are substantially larger, with female *P*. *hosii* reaching a maximum body size of 104.8 mm (males 79.6 mm) and female *P*. *rugosus* having body sizes up to 95.0 mm (males 76.8 mm). No adult specimens of *P*. *everetti* have ever been reported [3–5].

Several recent phylogenetic studies have demonstrated that Indian and Southeast Asian *Pedostibes* are polyphyletic, with the type species, *P*. *tuberculosus* being part of a clade comprising the South Asian toad genera such as *Duttaphrynus*, *Xanthophryne*, and *Adenomus*, whereas the Southeast Asian species are nested within a Southeast Asian radiation and is the sister lineage to the genus *Phrynoidis* [10–15]. Based on these results, we re-examined the taxonomic status of Southeast Asian *Pedostibes* to determine if their polyphyletic relationships, and presumed distinctiveness, can be corroborated with other lines of evidence. We demonstrate that in addition to being allopatric and polyphyletic with respect to the Indian taxa, the Southeast Asian species, *P*. *hosii* and *P*. *rugosus* (and presumably, by implication, also *P*. *everetti*) exhibit significant differences in morphology and reproductive strategies. The combination of these findings supports the hypothesis that the Southeast Asian and Indian species are not congeneric. Here, we define a new genus that better reflects the taxonomic placement and unique evolutionary history of the Southeast Asian taxa.

## Materials and Methods

### Morphological analysis

The following measurements (defined in [16]) were measured with a Mitutoyo digital caliper (to the nearest 0.1 mm) on the left side of the body: snout-vent length (SVL), snout length (SNL), head length (HL), head width (HW), tympanum diameter (TD), forearm length (FAL), third finger length (Fin3L), third finger disc width (Fin3DW), femur length (FL) tibia length (TBL), inner metatarsal tubercle length (MTTL). List of specimens examined are provided in S1 Table. For consistency, only male specimens of *Pedostibes tuberculosus*, *P*. *hosii* and *P*. *rugosus* were included in analyses (specimens of *P*. *kempi* and *P*. *everetti* were unavailable for examination). All measurements were corrected for proportional variance (due to large differences in body size) by individually dividing each character by the specimen’s SVL. Subsequent analyses were conducted on these adjusted measurements using the statistical software environment R v.3.1.2 [17]. Data were initially explored with simple mensural comparisons undertaken by plotting each character against its adjusted measurement. A principal component analysis (PCA) was then performed to find the best low-dimensional representation of morphological variation in the data and to further determine whether continuous morphological variation could form the basis of statistically detectable group structure. Principal components with eigenvalues of 1.0 or more were retained in accordance to Kaiser’s criterion [18]. To characterize clustering and distance in morphospace, a discriminant analysis of principal components (DAPC) was performed to find the linear combinations of morphological variables that have the largest between-group variance and the smallest within-group variance. DAPC relies on data transformation using PCA as a prior step to discriminant analysis (DA), ensuring that variables included in the DA are uncorrelated and number fewer than the sample size [19]. The DAPC analysis was performed using the R package “adegenet 2.0.0” [20].

### Phylogenetic analysis

We sampled one species from each major South and Southeast Asian bufonid genera that were shown from previous studies to form well-resolved, monophyletic clades [12, 14, 15]. Sequences for one mitochondrial (16S) and two nuclear markers (CXCR4, NCX1) were obtained from Genbank (Table 1) and aligned using the MUSCLE algorithm implemented in the program Geneious v 5.3.6 [21]. Open reading frames of protein-coding genes were manually inspected by eye and concatenated for subsequent analysis. The final concatenated alignment consisted of 3,375 base pairs, 711 patterns and 395 informative sites. We used the program PartitionFinder [22] to select the best-fit partitioning schemes and nucleotide substitution models under the Bayesian Information Criterion (Table 2). A partitioned maximum likelihood (ML) phylogenetic analysis was performed with the program RAxML [23] using the GTR + Γ nucleotide substitution model. Node support was assessed with 500 bootstrap replicates using the rapid hill-climbing algorithm. A Bayesian analysis was implemented in the program MrBayes 3.2.6 [24] using two independent runs (four chains each) with a MCMC chain length of 50,000,000 generations per run. Parameter and tree convergence were assessed using the program Tracer v.1.6 [25]. The MrBayes analysis was performed through the CIPRES Science Gateway [26]. Uncorrected pairwise p-distances were calculated in PAUP* [27] and visualized as a heatmap using R. Variation in male body size (SVL) was mapped onto the phylogeny using the R package ‘phytools’ [28].

**Table 1:** List of Genbank sequences used in phylogenetic analyses.

Species		Genbank #	
	16S	CXCR4	NCX1
*Adenomus kelaartii*	KM921789	EF107447	EF107221
*Anaxyrus boreas*	DQ158436	DQ306499	FJ882678
*Ansonia spinulifer*	AB435284	FJ882696	FJ882643
*Duttaphrynus melanostictus*	AY680268	KF665993	AY948805
*Ghatophryne ornata*	FJ882797	FJ882694	FJ882641
*Ingerophrynus divergens*	AB331715	FJ882701	FJ882648
*Pedostibes hosii* (Pen. Msia)	AY325993		
*Pedostibes hosii* (Borneo)	DQ283164	EF107449	EF107223
*Pedostibes rugosus*	AB331719		
*Pedostibes tuberculosus*	FJ882793	FJ882693	FJ882640
*Pelophryne signata*	AB746456	FJ882699	FJ882646
*Phrynoidis aspera*	AB530653	KF665952	
*Phrynoidis juxtaspera*	AB331713		FJ882656
*Sabahphrynus maculatus*	AB331718		
*Xanthophryne koynayensis*	FJ882782	FJ882691	FJ882638

**Table 2:** Best-fit partitioning schemes and nucleotide substation models under the Bayesian Information Criterion (BIC).

Subset Partitions	Best Model
cxcr4 (codon 1), ncx1 (codon 1)	HKY+I
cxcr4 (codon 2), ncx1 (codon 2)	F81+I
cxcr4 (codon 3), ncx1 (codon 3)	HKY+ Γ
16s	GTR+I+ Γ

### Nomenclatural Acts

The electronic edition of this article conforms to the requirements of the amended International Code of Zoological Nomenclature, and hence the new names contained herein are available under that Code from the electronic edition of this article. This published work and the nomenclatural acts it contains have been registered in ZooBank, the online registration system for the ICZN. The ZooBank LSIDs (Life Science Identifiers) can be resolved and the associated information viewed through any standard web browser by appending the LSID to the prefix "http://zoobank.org/". The LSID for this publication is: urn:lsid:zoobank.org:pub:1FE81DD3-BC81-4822-B50B-27240B125153. The electronic edition of this work was published in a journal with an ISSN, and has been archived and is available from the following digital repositories: PubMed Central, LOCKSS.

## Results

### Analysis of morphological variation

**Fig 2:**
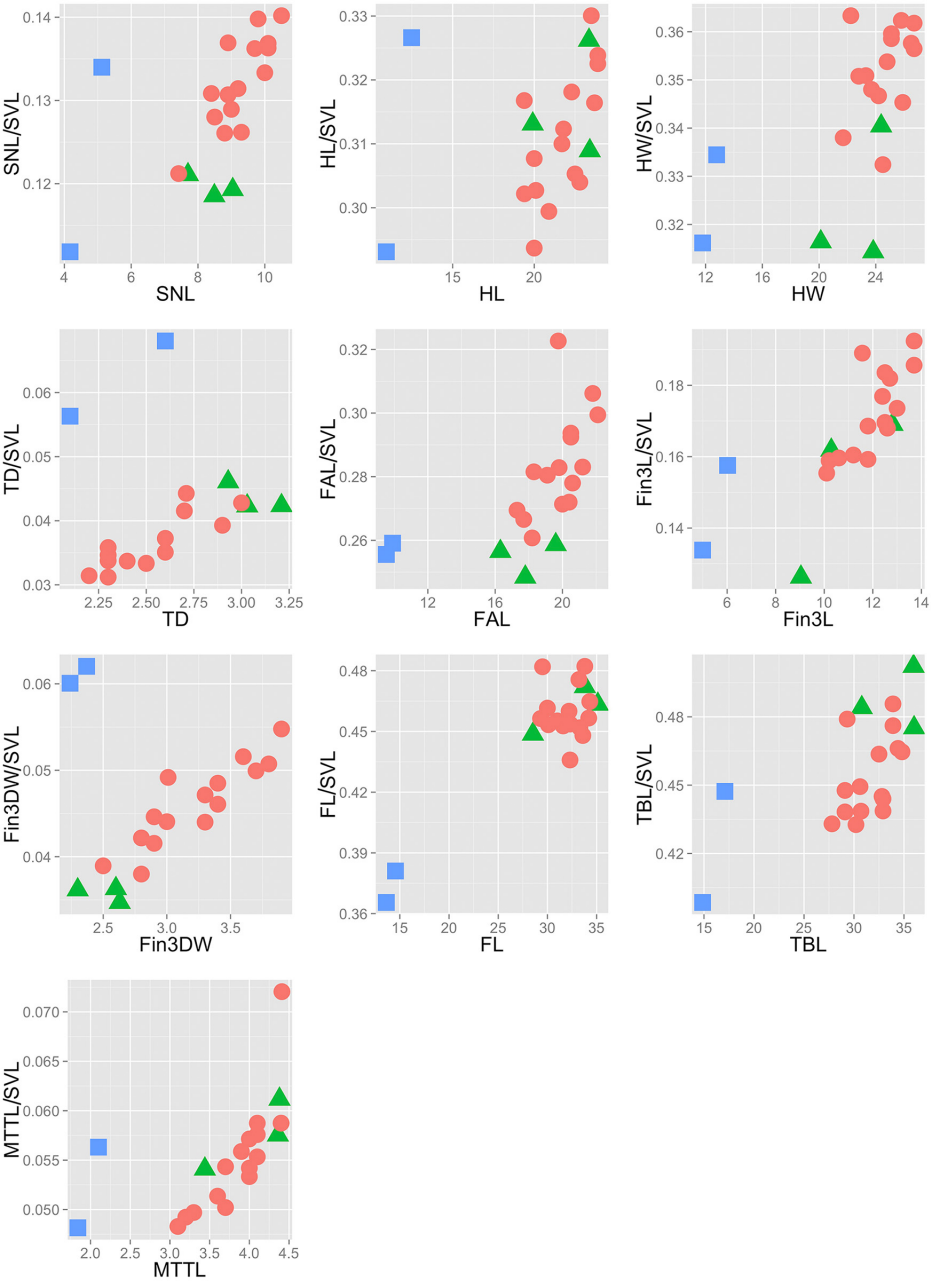
Scatterplots of continuous morphological characters against their adjusted measurements. Squares = *Pedostibes tuberculosus*; circles = *‘P’*. *hosii*; triangles = *‘P’*. *rugosus*

**Fig 3:**
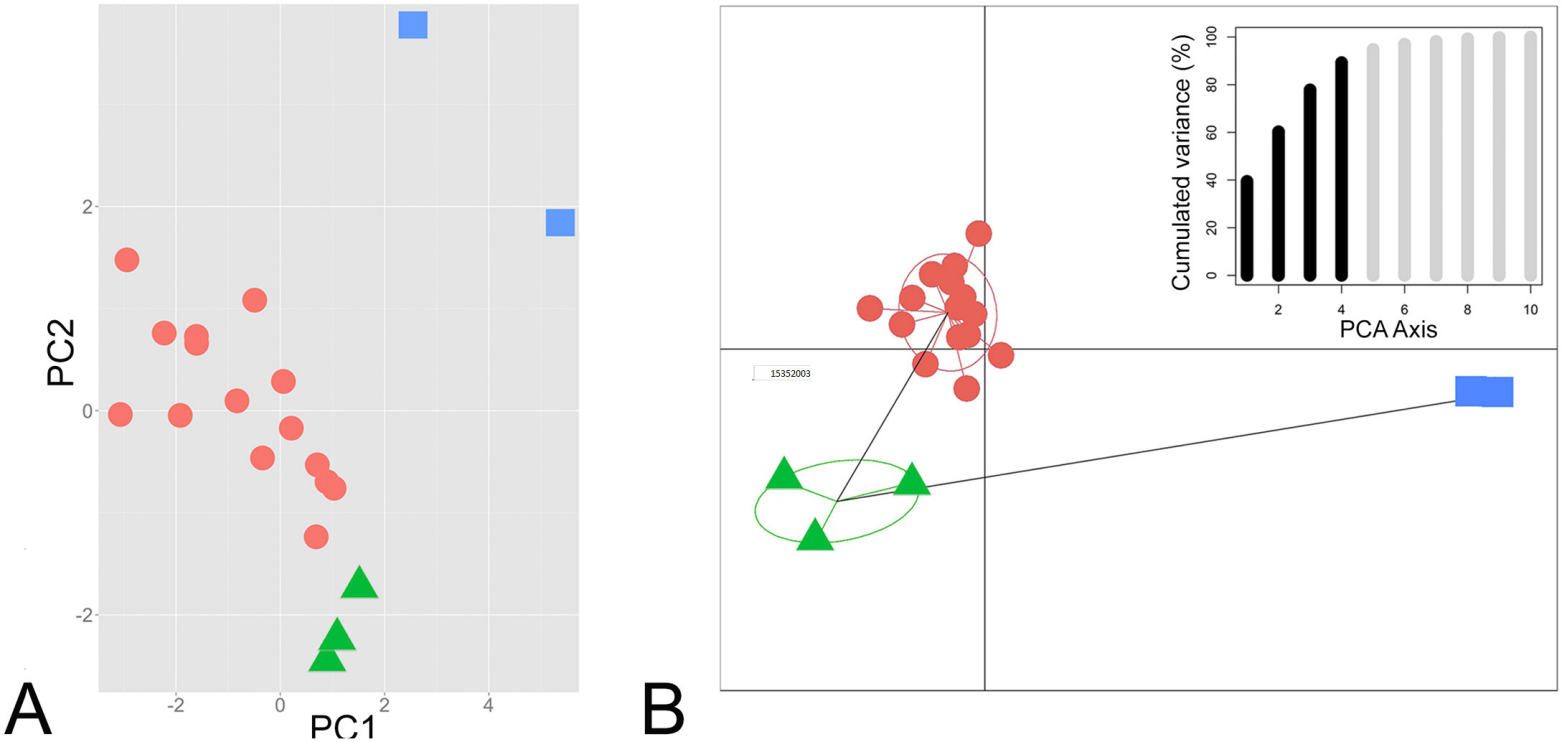
PCA and DAPC plots. (A) PCA plot for the first two principal components. (B) Scatterplot from the DAPC analysis. Lines from the centroid of the clusters represent a minimum-spanning tree based on the squared distances between species. Inset shows the cumulated variance along PCA axes. Squares = *Pedostibes tuberculosus*; circles = *‘P’*. *hosii*; triangles = *‘P’*. *rugosus*

Bivariate plots show clear and complete separation between *Pedostibes tuberculosus* and ‘*Pedostibes’ hosii* + *‘P’*. *rugosus* for every morphological variable (Fig 2). The first four principal components had eigenvalues of more than 1.0 and accounted for 90% of the total variance. These were retained for the DAPC analysis. The first principal component (PC1) had strong loadings on the characters HW, FAL, FL, and Fin3L, indicating that these characters explained most of the variation along the PC1 axis. The second component (PC2) possessed heavy loadings for Fin3DW and FL (Table 3). Ordination of the first two principal components shows complete separation between *P*. *tuberculosus* and *‘P’*. *hosii* + *‘P’*. *rugosus* along both axes (Fig 3A). Results of the DAPC analysis show exclusive clustering between all three species and a minimum-spanning tree based on the squared distances between populations demonstrate a substantial distance in morphospace between *P*. *tuberculosus* from India and *‘P’*. *hosii* + *‘P’*. *rugosus* from southeast Asia (Fig 3B).

**Table 3:** Summary statistics and loadings of the principal component analysis (PCA).

	PC1	PC2	PC3	PC4	PC5	PC6	PC7	PC8	PC9	PC10
Standard deviation	1.99	1.44	1.33	1.07	0.74	0.47	0.34	0.33	0.23	0.15
Proportion of Variance	0.39	0.21	0.18	0.11	0.05	0.02	0.01	0.01	0.01	0.00
Cumulative Proportion	0.39	0.60	0.78	0.89	0.95	0.97	0.98	0.99	1.00	1.00
Eigenvalues	3.95	2.08	1.76	1.14	0.55	0.22	0.12	0.11	0.05	0.02
Loadings:										
SNL/SVL	-0.27	0.31	-0.38	0.42	-0.04	0.02	-0.37	0.52	0.25	-0.18
HL/SVL	-0.24	0.19	0.42	0.53	-0.15	-0.35	0.47	0.11	-0.10	0.26
HW/SVL	-0.42	0.21	-0.13	-0.06	-0.49	0.28	-0.07	-0.24	-0.61	-0.12
TD/SVL	0.27	0.32	0.43	0.11	0.31	0.64	-0.03	0.26	-0.23	0.00
FAL/SVL	-0.43	0.20	0.02	-0.29	0.17	0.22	0.54	-0.04	0.39	-0.40
Fin3L/SVL	-0.40	0.10	-0.11	-0.19	0.70	-0.28	-0.13	0.05	-0.39	0.21
Fin3DW/SVL	0.00	0.65	0.14	-0.19	-0.09	-0.08	-0.30	-0.38	0.36	0.37
FL/SVL	-0.38	-0.41	-0.05	0.08	-0.01	0.48	-0.02	0.00	0.24	0.63
TBL/SVL	-0.28	-0.26	0.46	0.32	0.17	-0.01	-0.42	-0.40	0.12	-0.39
MTTL/SVL	-0.21	-0.13	0.49	-0.51	-0.29	-0.16	-0.24	0.53	0.02	-0.01

### Phylogenetic analysis

**Fig 4:**
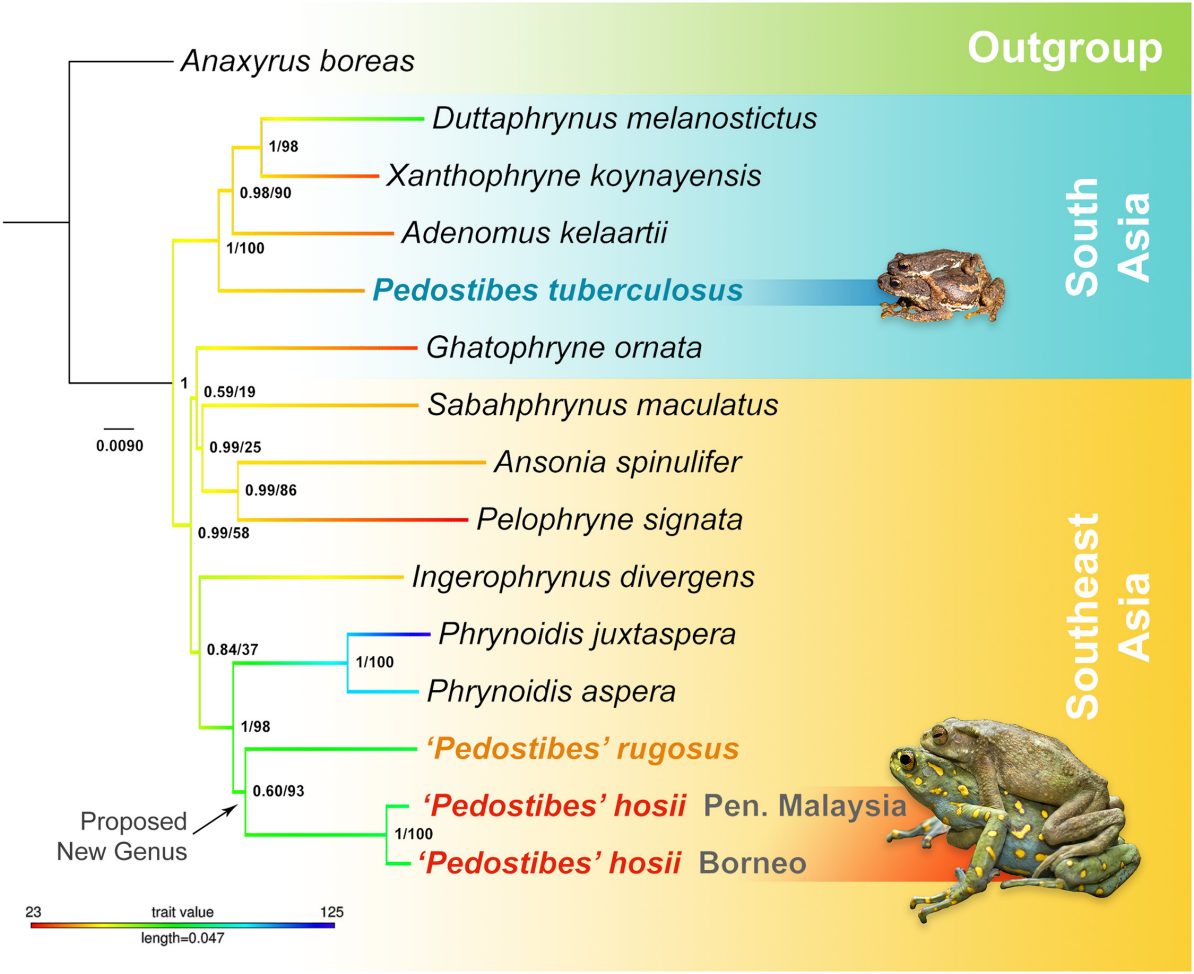
Maximum likelihood phylogeny showing the polyphyletic relationship of *Pedostibes* and the proposed new genus among the South & Southeast Asian Bufonids. Node support values are Bayesian posterior probabilities followed by maximum likelihood bootstrap values. Trait values denote maximum male SVL in mm. Images of specimens are scaled to relative body size.

**Fig 5:**
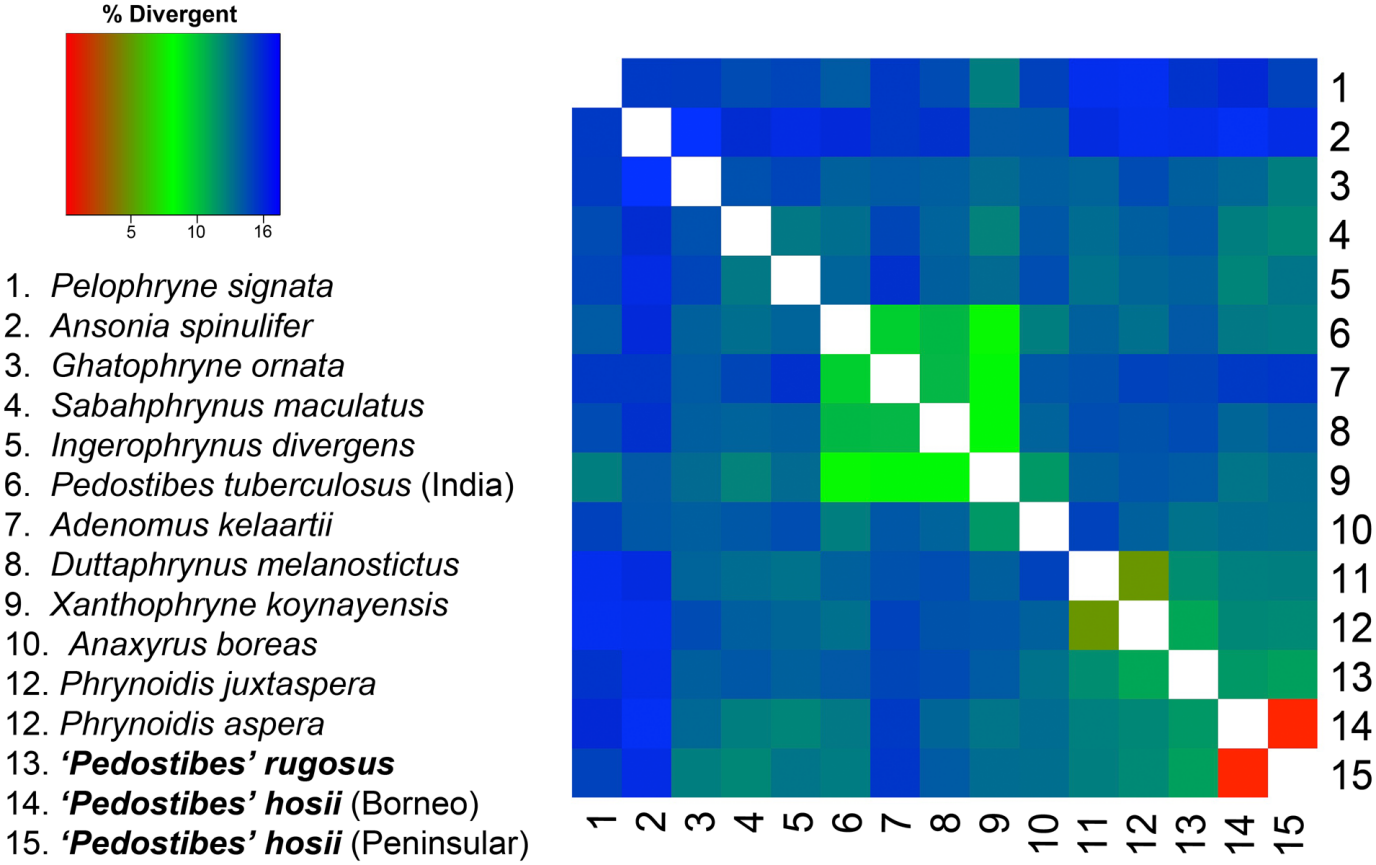
Heatmap of pairwise uncorrected p-distances. The colors red to blue indicate low to high divergences.

Both ML and Bayesian phylogenies recovered similar topologies, with the ML tree providing better resolution at deeper nodes. *Pedostibes tuberculosus* was recovered as part of a primarily South Asian monophyletic clade, where it forms a sister lineage relationship to the clade that contains the genera *Adenomus*, *Xanthophryne*, and *Duttaphrynus* (*Pedostibes* + (*Adenomus* + (*Xanthophryne* + *Duttaphrynus*))). Males from this clade tend to be small in size with the exception of the genus *Duttaphrynus*. Southeast Asian *‘Pedostibes’* were reciprocally monophyletic with the genus *Phrynoidis* and represent a clade that exhibits the largest body size among all South and Southeast Asian toads (Fig 4; S2 Table). Uncorrected p-distances calculated from the mitochondrial alignment demonstrate that Southeast Asian *‘Pedostibes’* are 13–14% divergent from *P*. *tuberculosus* and 12–13% divergent from the genus *Phrynoidis*. These levels of divergences are consistent with other generic level divergences among South Asian, and Southeast Asian bufonids (Fig 5; S3 Table).

### Natural history

**Fig 6:**
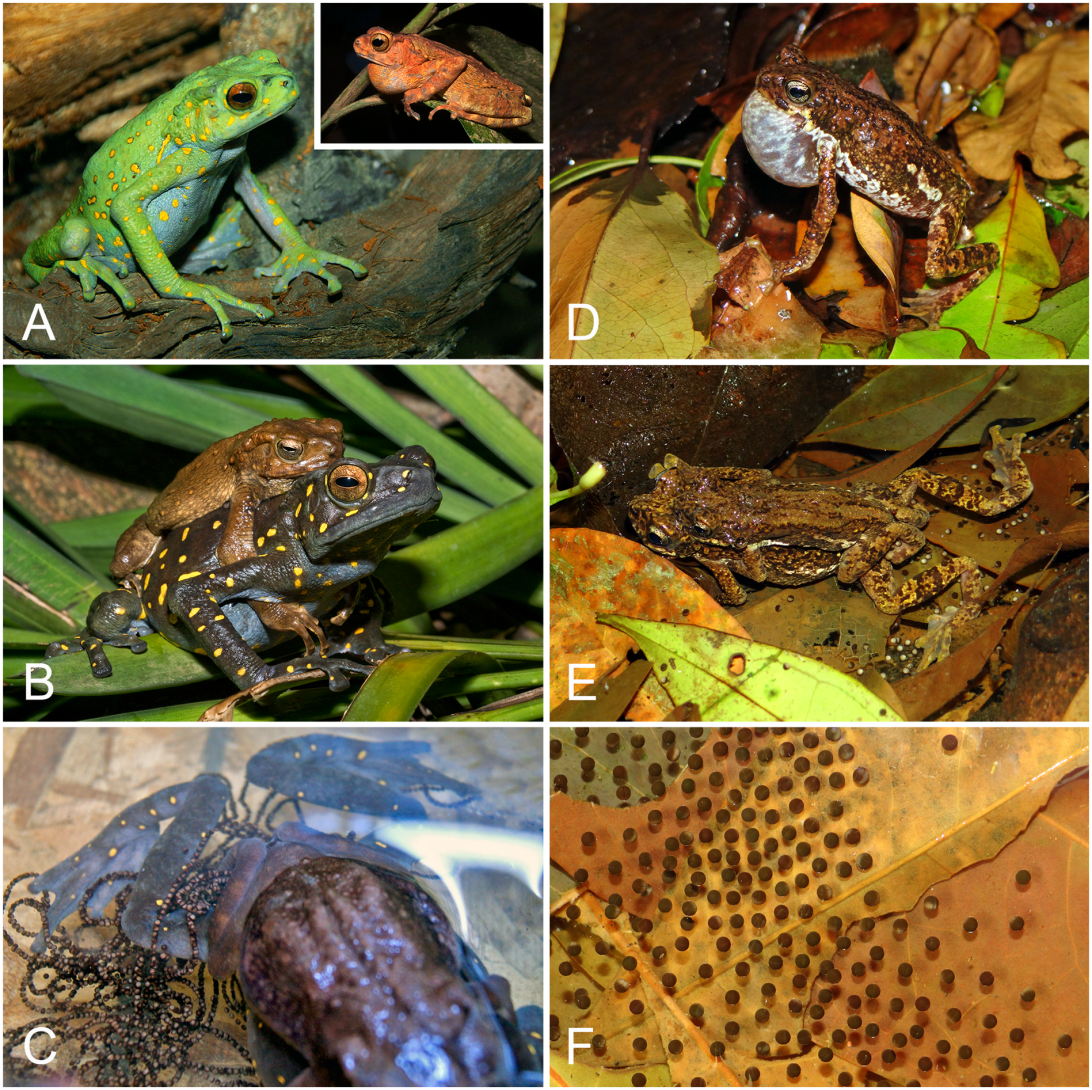
Color pattern and reproductive comparisons between *Pedostibes tuberculosus* from India and ‘*P’*. *hosii* from Southeast Asia. (A) *‘Pedostibes’*. *hosii* female from Peninsular Malaysia; inset = vocalizing male from Borneo. (B) Amplectic pair of ‘*P’*, *hosii* from Peninsular Malaysia. (C) ‘*P’*. *hosii* from Peninsular Malaysia showing oviposition of clutch as strings. (D) Vocalizing male of *P*. *tuberculosus* from India. (E) Amplectic pair of *P*. *tuberculosus* engaged in oviposition. (F) Film of eggs of *P*. *tuberculosus* floating in a stream pool. Fig 6C reprinted from JoshFrogs.com under a CC BY license, with permission from JoshFrogs, original copyright 2014.

Both South Asian and Southeast Asian *Pedostibes* are forest stream-associated taxa. Males vocalize (Fig 6D) from low foliage, elevated banks or branches of mid-story riparian vegetation along small to medium-sized forest streams. ‘*Pedostibes’ hosii* is primarily a lowland frog (20–525 m a.s.l; [3]), while *P*. *tuberculosus* is a mid- to high-elevation species (300–1400 m a.s.l). The breeding activity of *P*. *tuberculosus* commences in the pre-monsoon season between late April and late May. Females respond to vocalizing males by approaching their perch. Axillary amplexus is assumed (Fig 6E), after which the amplectic pair descends to the relatively dry streambed, where oviposition occurs in stagnant, rocky pools. Pigmented eggs are laid as films [29] with an average of ~1100 eggs/clutch, which float beneath the surface of the water in the pool (Fig 6F). Each egg is of an average of 1.1 mm in diameter. For *‘P’*. *hosii*, breeding is similar to *P*. *tuberculosus* in that amplexus is axillary (Fig 6B). However, the oviposition mode is different in that the eggs are laid as long strings (Fig 6C) with an average of ~4000 eggs/clutch, typical of many other large bodied bufonids [30]. Egg size is approximately 1.2 mm in diameter. Reproductive behavior of *P*. *kempi*, *‘P’*. *rugosus* and ‘*P’*. *everetti* are unknown.

### Systematics

Distribution, morphological, natural history, phylogenetic relationships and genetic divergences and genetic data do not support the current taxonomy that unites South Asian and Southeast Asian tree toads under the common genus *Pedostibes*. Because the Indian species *P*. *tuberculosus* has priority as the type species, we propose that the Southeast Asian lineages (‘*P’*. *hosii*, and *‘P’*. *rugosus*) be recognized as a separate genus. One remaining, unresolved issue concerns the generic status of *P*. *kempi* and *‘P*.*’ everetti*. *Pedostibes kempi* has not been collected since its original description and no modern specimens or genetic samples are available for study. ‘*Pedostibes’ everetti* is phenotypically similar to ‘*P’*. *rugosus* and the only discrete morphological character differentiating *‘P’*. *everetti* from ‘*P’ rugosus* is the absence (vs. presence) of a tarsal ridge [3]. Furthermore, ‘*P’*. *everetti* is only known from one juvenile specimen. Based on these limited data, the taxonomic status of these species remains uncertain and additional data will be required to resolve their taxonomic placement. Thus, based on distribution, we tentatively assign *P*. *kempi* to the genus *Pedostibes* and *‘P*.*’ everetti* to the new genus named below, with the caveat that these relationships may need to be re-evaluated when additional data become available. Because the earliest species description of the Southeast Asian group is ‘*P*.*’ hosii*, and its only generic synonym, *Nectophryne* is occupied by a distinct African tree toad lineage [14,31], we define and diagnose a new genus for the Southeast Asian clade as follows:

### Description of New Genus

*Rentapia* **gen. nov.** urn:lsid:zoobank.org:act:B692321E-96EF-496E-973B-2348FF22764C

Type species: *Nectophryne hosii* Boulenger, 1892; Holotype: BMNH 92.6.3.19

#### Diagnosis

Large-bodied toads with a movable coccyx, eight pre-sacral vertebrae, a complete quadratojugal, and small, pigmented ova [3,8] laid as strings. Interorbital cranial crests absent. Parotoid glands large, distinct; oval, circular or triangular in dorsal view. Fingers webbed at base, tips expanded into flat discs. Feet fully webbed on all toes except fourth. Nuptial pads present in males. Adults are primarily arboreal and inhabit riparian vegetation around small- to moderately-sized forest streams.

#### Phylogenetic definition

*Rentapia* is a node-based name that refers to the clade originating from the most recent common ancestor of ‘*Pedostibes*’ *(Rentapia) hosii* and ‘*P*.’ *rugosus* (Fig 4).

#### Content

The allopatric species of *Pedostibes* of Southeast Asia from the Isthmus of Kra, Thailand and Peninsular Malaysia, Sumatra, and Borneo: *Rentapia hosii* and *R*. *rugosus*. We also tentatively place *‘P*.*’ everetti* in *Rentapia* because of its close geographic proximity with the two Southeast Asian taxa, as well as the character state similarities it shares with *R*. *rugosus* (webbed hands and feet, finger and toe tips dilated into truncate disks, movable coccyx, eight pre-sacral vertebrae, complete quadratojugal).

#### Etymology

The Iban are a subgroup of the indigenous peoples of Borneo (collectively known as the Dayaks) and form the main ethnic group in the Malaysian state of Sarawak. The generic epithet is selected to honor the legendary Iban warrior Libau Rentap, a great war chief, freedom fighter, and Malaysian national hero. Renowned for headhunting, the Iban were subjugated in Sarawak by the White Rajahs (English monarchy), who sought to confiscate land and impose taxes. Libau Rentap rose against the first of the White Rajahs, James Brooke, initiating a rebellion that eventually returned power to the people of Sarawak. The rule of the White Rajahs lasted from 1841 to 1946.

## Discussion

*Pedostibes* is small in size and deposits medium-sized clutches of eggs that are laid as films. The combination of these traits differentiates it from *Rentapia* **gen. nov.**, which is diagnosed by its larger body size and an oviposition mode characterized by a large clutch of eggs laid as strings. Given that individual egg sizes in both genera are comparatively similar, this agrees with past studies that show a close correlation of clutch size to female body size [32]. There are also differences in sexually dimorphic traits. Both male and female *Pedostibes* have the same color pattern, whereas *Rentapia hosii* females are vividly colored with bright markings, as compared to the duller, uniformly colored males (Fig 6A–6C). However, this color dimorphism has so far been observed only in *R*. *hosii* and we do not have evidence for either *R*. *rugosus* or *R*. *everetti* having such color dimorphism.

The most widespread *Rentapia*, *R*. *hosii* exhibits marked geographically based variation in female color pattern at different localities across its known distribution. Our analyses inferred a strongly supported genetic split between *R*. *hosii* populations from Peninsular Malaysia and those from Borneo. Additionally, females from these populations are phenotypically distinct. Females from Peninsular Malaysia are light green (dark gray when handled or stressed) with large, sparse, irregular yellow spots (Fig 6A–6C), whereas females from Borneo are purplish with yellow vermiculation or uniformly brown. Furthermore, a population from Danum Valley, Sabah, exhibits a phenotype that is distinct from populations from Peninsular Malaysia and the rest of Borneo. Females from Danum Valley have more dense dorsal vermiculations and broad, light-colored marbling on the flanks and posterior region of the thigh. The venter shows faint but distinct marbling whereas the venters of the former two populations are immaculate. However, the genetic divergences between Peninsular Malaysian and Bornean populations are low (2.6%) and more data are required to determine whether these populations represent distinct species.

Since several recent studies have shown *Pedostibes* **sensu lato** to be a non-monophyletic genus within the family Bufonidae, [13] suggested uniting *Rentapia* with its sister genus, the terrestrial river toad *Phrynoidis*, as a taxonomic solution. Although this suggestion would resolve the issue of monophyly, we find the recognition of the new genus to be a preferable solution based on several lines of reasoning. First, *Rentapia* and *Phrynoidis* exhibit striking morphological differences: *Rentapia* has expanded and flat finger discs (dilated into keratinized, bulbous tips in *Phrynoidis*); is considerably smaller in size (female *Phrynoidis* range from 120 mm to more than 200 mm SVL; [3]); lacks supernumerary palmar tubercles (present in *Phrynoidis*); possesses basal interphalangeal finger webbing (webbing absent in *Phrynoidis*); and the oral disk of *Rentapia* tadpoles is half the maximum width of the body [3] (extends the entire width of the body in *Phrynoidis* [5]). Striking ecological differences are equally apparent. *Rentapia* is an arboreal habitat specialist, whereas *Phrynoidis* are terrestrial riparian habitat generalists [3], that oviposits enormous clutches with an average size of 12,792 eggs per clutch [33]; Finally, we note that genetic divergences of >12.0% (Fig 5) is consistent with other inter-generic divergences within Bufonidae [14,15], suggesting that our proposition is not discordant with other, accepted taxonomic arrangements. Although merging *Rentapia* and *Phrynoidis* would remedy the polyphyly of *Pedostibes* **sensu lato**, the preponderance of differences in morphology, reproductive characteristics, ecology, and molecular characters is best acknowledged by considering these two lineages as separate genera.

## Supporting Information

S1 Appendix Copyright permission for Fig 1. (DOCX)

S2 Appendix Copyright permission for Fig 6C. (DOCX)

S1 Fig Maximum likelihood phylogeny. Node support values represent bootstrap support values.
(PDF)

S2 Fig Bayesian phylogeny. Node support values represent posterior probabilities.
(PDF)

S1 Table Specimens examined. Museum abbreviations: FMNH = Field Museum of Natural History, Chicago; TNHM = Natural History Museum, Trivandrum, India.
(XLSX)

S2 Table Maximum male and female SVL. [34–36]
(XLSX)

S3 Table Pairwise uncorrected p-distances calculated from the 16S mitochondrial gene. (XLSX)
