# Supplementary material for: Polyphyly of Asian Tree Toads, Genus Pedostibes Günther, 1876 (Anura: Bufonidae), and the Description of a New Genus from Southeast Asia
Source: PLoS One. 2016 Jan 20;11(1):e0145903. doi: 10.1371/journal.pone.0145903 (PMC4720419; doi:10.1371/journal.pone.0145903)
Supplement: S1 Appendix — (DOCX) [file pone.0145903.s001.docx]

Begin forwarded message:

**From:**"Treehouse Maps" <[maps@treehouse-maps.com](mailto:maps@treehouse-maps.com)>

**Date:**November 25, 2015 at 06:43:51 CST

**To:**"Robin Abraham" <[robinabrahamf50@gmail.com](mailto:robinabrahamf50@gmail.com)>

**Subject: Re: Permission to use Shaded Relief Map**

Hi Robin,

Thank you for your email.

This reply gives you explicit written permission to use your modified version of the map, which may also be used by any other parties, in any way you like.

I am intrigued by your map. Clearly there is going to be something medically helpful about it. This is beyond my knowledge, but I should explain that I am in fact a recently-retired water resources engineer who, since the 1970s, has spent most of his career in the area covered by your map. A nice coincidence!

Feel free to spend me a link to your publication when ready (no hurry) just to satisfy my curiosity!

Best regards,

Paul at Treehouse Maps

**From**: "Robin Abraham" <[robinabrahamf50@gmail.com](mailto:robinabrahamf50@gmail.com)>
**Sent**: Tuesday, November 24, 2015 9:49 AM
**To**: [maps@treehouse-maps.com](mailto:maps@treehouse-maps.com)
**Subject**: Re: Permission to use Shaded Relief Map

Hi Paul,

I had contacted you a few weeks ago seeking permission to use your map in an open access peer-reviewed publication. As per protocol, I would need your written consent in the following format to have the figure accepted for use under Creative Commons;

“**I request permission for the open-access journal PLOS ONE to publish XXX under the Creative Commons Attribution License (CCAL) CC BY 4.0 (**[**http://creativecommons.org/**](http://creativecommons.org/licenses/by/4.0/)**). Please be aware that this license allows unrestricted use and distribution, even commercially, by third parties. Please reply and provide explicit written permission to publish XXX under a CC BY license.”**
