# Supplementary material for: Polyphyly of Asian Tree Toads, Genus Pedostibes Günther, 1876 (Anura: Bufonidae), and the Description of a New Genus from Southeast Asia
Source: PLoS One. 2016 Jan 20;11(1):e0145903. doi: 10.1371/journal.pone.0145903 (PMC4720419; doi:10.1371/journal.pone.0145903)
Supplement: S2 Appendix — (DOCX) [file pone.0145903.s002.docx]

| \| 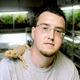 \| **Zach** (Josh's Frogs HelpDesk)  Oct 2, 3:54 PM  Hi Chan,  As long as you credit the photos to JoshsFrogs.com, you're more than welcome to use any images off of the website. We'd love to read your article once it's finished! \| \| --- \| --- \| |
| --- | --- | --- |

| \| 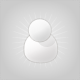 \| **Kin Onn**  Oct 1, 5:03 PM  Dear Josh Frogs,  I'm Chan Kin Onn, a graduate student at the University of Kansas. I'm currently writing a paper on the natural history of Pedostibes hosii and was wondering if it was possible to request permission to use some of your photos, namely the ones showing the frogs in amplexus with strings of eggs. You will of course be duly credited and acknowledged. Thank you for your time!  Regards,  Chan \| \| --- \| --- \| |
| --- | --- | --- |
