# Supplementary figures and images for: Polyphyly of Asian Tree Toads, Genus Pedostibes Günther, 1876 (Anura: Bufonidae), and the Description of a New Genus from Southeast Asia
Source: PLoS One. 2016 Jan 20;11(1):e0145903. doi: 10.1371/journal.pone.0145903 (PMC4720419; doi:10.1371/journal.pone.0145903)

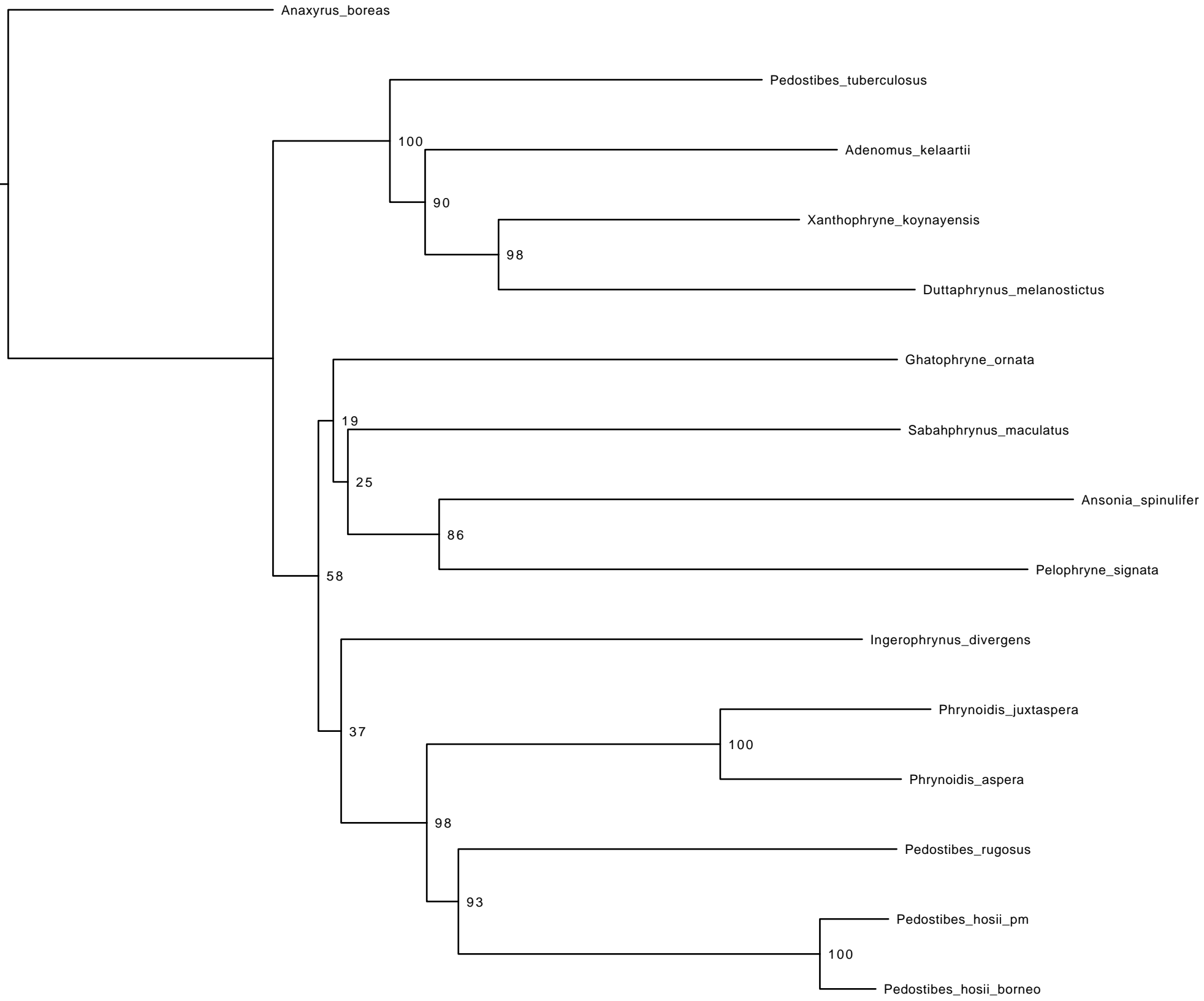

0.0090

Supplement: S1 Fig — Node support values represent bootstrap support values. (PDF) [file pone.0145903.s003.pdf]

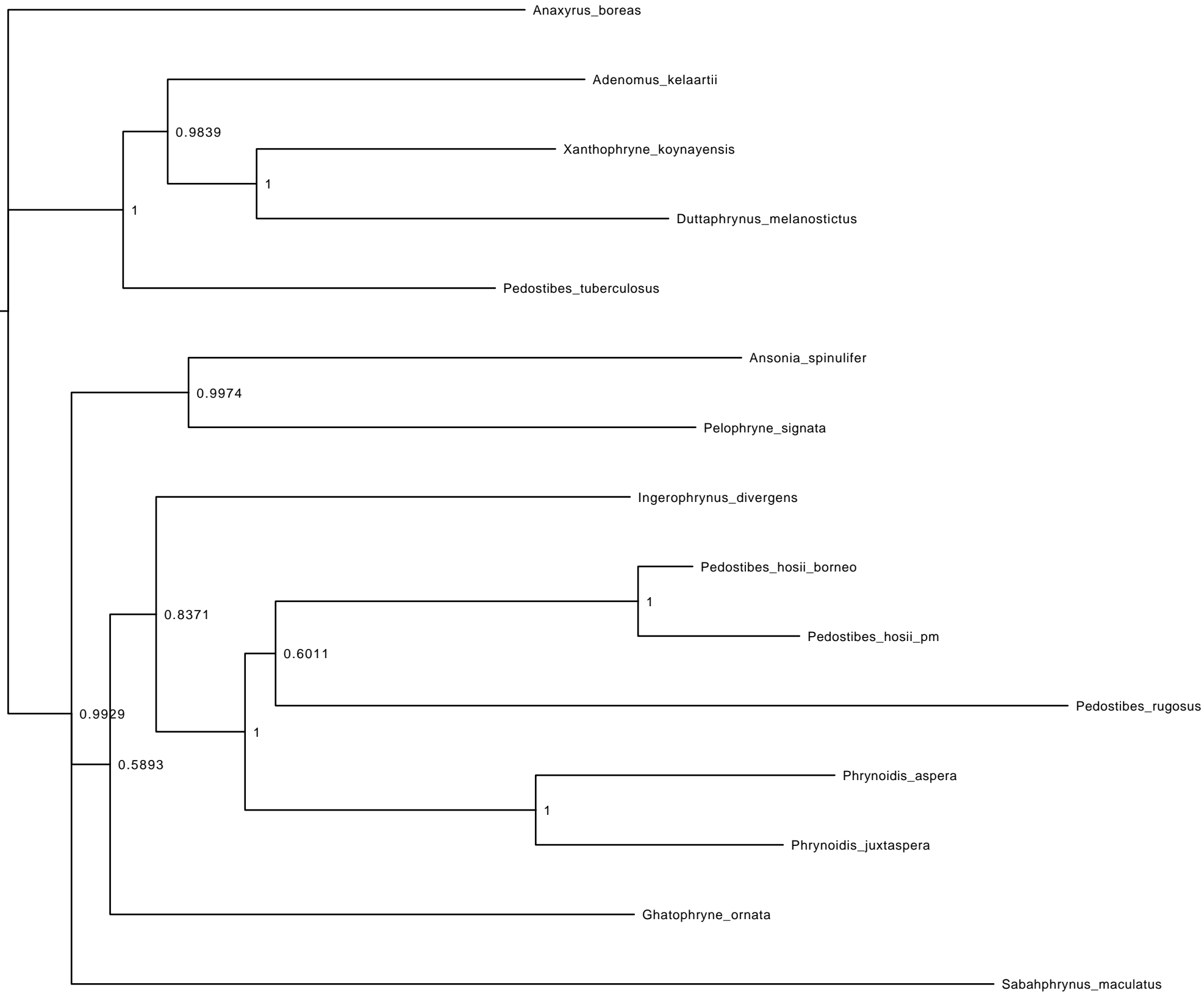

0.0080

Supplement: S2 Fig — Node support values represent posterior probabilities. (PDF) [file pone.0145903.s004.pdf]
